# Secondary Electron Emission Materials for Transmission Dynodes in Novel Photomultipliers: A Review

**DOI:** 10.3390/ma9121017

**Published:** 2016-12-16

**Authors:** Shu Xia Tao, Hong Wah Chan, Harry van der Graaf

**Affiliations:** 1 Department of Applied Physics, Eindhoven University of Technology, Eindhoven 5600 MB, The Netherlands; 2 Center for Computational Energy Research, Dutch Institute for Fundamental Energy Research (DIFFER), Eindhoven 5600 HH, The Netherlands; 3 Department of Microelectronics, Delft University of Technology, Delft 2600 AA, The Netherlands; H.W.Chan@tudelft.nl (H.W.C.); vdgraaf@nikhef.nl (H.v.d.G.); 4 National Institute for Subatomic Physics (Nikhef), Science Park 105, Amsterdam 1098 XG, The Netherlands

**Keywords:** photon detector, photomultiplier, transmission dynode, secondary electron emission, secondary electron yield, atomic layer deposition

## Abstract

Secondary electron emission materials are reviewed with the aim of providing guidelines for the future development of novel transmission dynodes. Materials with reflection secondary electron yield higher than three and transmission secondary electron yield higher than one are tabulated for easy reference. Generations of transmission dynodes are listed in the order of the invention time with a special focus on the most recent atomic-layer-deposition synthesized transmission dynodes. Based on the knowledge gained from the survey of secondary election emission materials with high secondary electron yield, an outlook of possible improvements upon the state-of-the-art transmission dynodes is provided.

## 1. Introduction

Secondary emission in physics is a phenomenon where primary incident particles of sufficient energy, when hitting a surface or passing through some material, induce the emission of secondary particles. The term Secondary Electron Emission (SEE) often refers to the emission of low energy electrons when high energy electrons in a vacuum tube strike a surface of an emission material; these are called secondary electrons. SEE was the subject of intense experimental study for a period of 20–30 years from the early 1930s, resulting in a vast amount of publications on this topic (including no less than six full-length books on the topic [1]). The aim of most of the work was to explore the mechanism of SEE, i.e., attempting to demonstrate the “universal law” [2] (the secondary electron yield versus primary energy curve). Materials studied in those literatures cover various types of materials (single elements, compounds, metals, semiconductors, and insulators) for a wide variety of applications. The focus of this review is to search for materials with high Secondary Electron Yield (SEY) for potential use in novel transmission dynodes for Photo Multipliers Tubes (PMTs). The scope will therefore be limited in two aspects. First, the paper will deal only with materials that have high SEY. Second, in terms of device configuration, the discussion will be limited to transmission dynodes, i.e., excluding reflective dynodes.

The structure of the paper is as follows. First, the working principles of PMTs and dynodes will be described (Section 2). Second, the definition of Reflection Secondary Electron Yield (RSEY), Transmission Secondary Electron Yield (TSEY) and the SEE mechanism will be described. (Section 3). The most promising candidates with high RSEY (>3) are tabulated for easy reference (Section 4). Then, generations of dynodes and their TSEY (>1) will be reviewed with detailed analysis of a few important materials, such as diamond and Atomic Layer Deposition (ALD) synthesized Al_2_O_3_ (Section 5). Finally, based on the knowledge gained in the previous sections, new concepts of the tunable material properties, ALD process, and device structure will be proposed for future development (Section 6).

## 2. The Basics of the PMTs

PMs are typically constructed with an evacuated glass housing, containing a photocathode, several dynodes, and an anode. The first PMT was invented in 1934 as a sensitive, low-noise, and fast light detector. PMTs are used extensively in scintillating detectors in nuclear physics, particle physics, astronomy, medical diagnostics, and security devices. They have been perfected during their 70 years of existence: the quantum efficiency of the photocathode has now reached a level of 43% at 350 nm [3], close to the theoretical maximum. Besides the quantum efficiency of the photocathode, performance is also affected by the transmission of the window material that the light passes through, by the materials, and by the arrangement of the dynodes.

**Figure 1:**
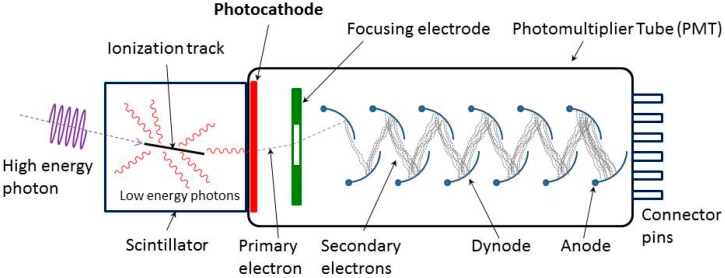
Working principle of a typical PMT [7]. A photon (ultraviolet, visible, or near-infrared light) is converted in the photocathode into a low-energetic electron, emitted into the vacuum. This electron is accelerated towards and focused onto the first dynode, releasing secondary electrons. This multiplication is repeated in subsequent dynodes, resulting in a measurable electric charge at the anode.

**Figure 2:**
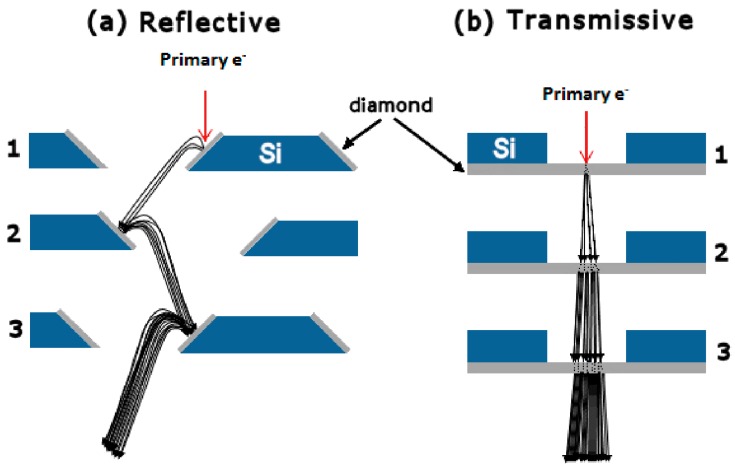
Two geometries (**a**) reflection and (**b**) transmission typically used for secondary electron multiplication dynode devices. Here, thin diamond film is used as the dynode material. Three multiplication stages are shown for each, with each stage having a higher positive bias compared to the previous stage. The figure is reproduced from reference [6].

In a PMT, dynodes are electrodes in a vacuum tube that serve as an electron multiplier through SEE (see Figure 1). The dynodes are so arranged that the electric fields between them cause the electrons emitted by each dynode to strike the next with an energy of a few hundred eV. As a result of secondary emission, the number of electrons increases from dynode to dynode, giving the required multiplication. With new coatings, the SEY of dynodes has greatly improved, reducing the required number of dynodes and their size [4]. The gain of each dynode and the overall gain of the multiplier fluctuates around a statistical mean. Gain fluctuations can be reduced by increasing the SEY, improving the uniformity of the SEY, and equalizing the collection efficiencies of the dynodes [5]. Important factors governing the gain and time response of the multipliers are the geometry and materials of the dynodes. The geometry of the dynodes can be a reflection or a transmission configuration. A large number of PMT models are available utilizing various combinations of design variables. The most commonly used models are based on a reflection configuration. Research and production of prototypes in transmission configuration is mainly carried out in laboratories. Figure 2 shows a direct comparison of diamond dynodes with a reflection and a transmission configuration [6]. The fundamental difference between these two configurations are the thickness of the dynodes. In reflection configuration, the thickness of the emission materials usually is in the order of µm or larger. However, in transmission configuration, much thinner dynodes are required to obtain a high TSEY.

The use of the transmission SEE as opposed to reflection SEE for electron multiplication has been proposed by Lubszynski [8], McGee [9] and Sternglass [10] in the mid-60s. The principal reason for this has been the short dynode lifetime due to the higher average currents which a particle-counter must be able to handle in the last few stages. The radiation damage caused by high energetic electron bombardment was expected to be reduced in a transmission configuration because the high energetic electrons would be slowed down after passing through the dynodes, converted to transmitted secondary electrons. Another important advantage of transmission dynodes is that they can avoid the feedback of the charged particles from the latter stage to cathode as well as to the back side of the dynodes with very often fragile surface terminations with a Negative Electron Affinity (NEA). Sternglass and Goetze described several other advantages of dynodes making use of the transmitted SEE [11]. These include (i) improvement in quantum detection efficiency and pulse amplitude fluctuations; (ii) a reduction in transit-time fluctuations; (iii) uniform response over a large area, eliminating transit-time differences from different parts of the cathode; (iv) ability to operate in the presence of intense magnetic fields when oriented along the axis of the tube; and (v) short total transit-time and dead-time.

## 3. SEY and SEE Mechanism

The electron–matter interactions determining SEE in both transmission and reflection can be described by three processes [12]: generation, transport, and escape. They are, respectively, (i) the production of internal secondary electrons by kinetic impact of the primary electrons; (ii) transport of the internal secondary electrons through the sample bulk toward the surface; and (iii) escape of the electrons through the solid–vacuum interface.

In the process of generation, the primary electrons slow down through collisions with electrons and ions and transferring kinetic energy to internally generated secondary electrons. At high primary energies, the high-velocity electrons have a relatively short time to interact with the lattice electrons, and the internal yield per unit length is low. As the primary electrons lose energy, the interaction time increases and so does the yield. The combined effect is that, as the primary-electron energy increases, the internal secondary electrons originate deeper beneath the surface.

In general, a primary electron with a reasonably high energy generates many internal secondary electrons. However, high SEYs are not always observed because most of the internal secondary electrons generated by deep absorption lose most of their energy through many scattering processes during the transport process. The energy-loss mechanisms for internal secondary electrons differ in metals and insulators. In metals, the secondary electrons lose energy by interacting with conduction electrons, lattice vibrations, and defects. The kinetic energy of a secondary electron must be at least fermi level plus work function (typically >10 eV) when it reaches the surface in order to escape. This large minimum escape energy and the high collision probability due to the large number of conduction electrons result in the low SEYs (usually <1) found with metals. In semiconductors and insulators, the lifetime of the secondary electrons depends, among other things, on the scattering processes (electron–electron scattering, electron–impurity scattering, electron–phonon scattering and so on) that can occur. Since there are few conduction electrons in insulators, the secondary electrons lose energy through the excitation of valence electrons into the conduction band. The wide band gap prevents secondary electrons with kinetic energy less than band gap energy from participating in such electron–electron collisions [12], significantly increasing their travel distance compared to metals. Therefore, in general, the SEYs are high in insulators. It should be noted that the number of initial secondaries produced varies inversely with the band gap, so improved transport comes at the cost of lower initial generation.

**Figure 3:**
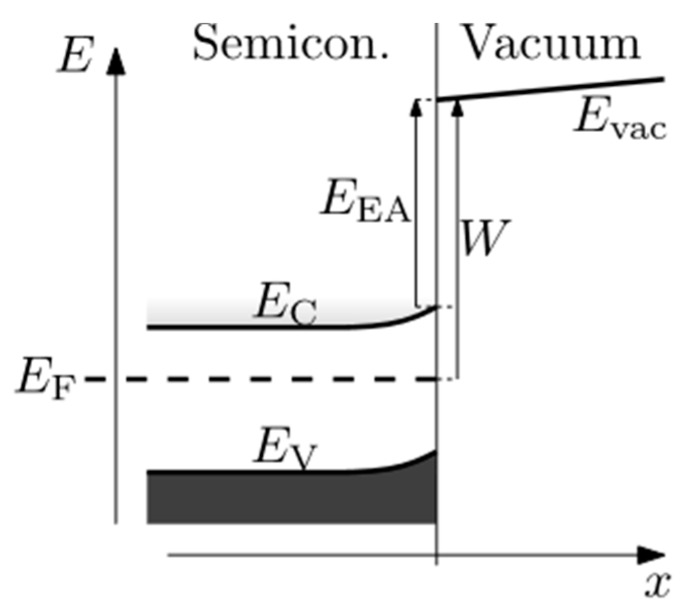
Band diagram of semiconductor-vacuum interface [13] showing electron affinity *E*_EA_, defined as the difference between near-surface vacuum energy *E*_vac_, and near-surface conduction band edge *E*_C_. Fermi level *E*_F_, valence band edge *E*_V_, work function *W*.

At the solid–vacuum interface, the thermalized electrons have to overcome a potential barrier (see Figure 3) called work function for metals and Electron Affinity (EA) for semiconductors and insulators. In metals, only few of the thermalized electrons will be able to cross the gap, due to the large work function (a few eV). In semiconductors and insulators, when in the presence of a positive EA, the majority of the secondary electrons will be trapped. However, in case of a NEA (when the *E*_vac_ lies lower than *E*_c_ in Figure 3 [13]) the secondary electron can be emitted into the vacuum freely. NEA can be achieved by certain surface modifications, for example, H termination on diamond or Cs and Cs-O terminations on semiconductors. In summary, high SEE materials are often insulators because they possess a large band gap as well as a relatively low EA compared to the work function of metals.

To simplify, the whole SEE process can be described as the transformation of a high-energy, low current electron beam into a low-energy, high-current secondary beam [14]. In this context, the secondary electron yield (SEY) is defined as the ratio of the current of emitted secondary electrons (<50 eV) to the current of incident primary electrons. As shown in Figure 2, when the thickness of the material is thin enough, the primary electrons are able to penetrate the material. In this case, SEE occur both on the front and back sides of the thin materials. The TSEY (the ratio of transmitted secondary current to the incident primary current) is used to distinguish the transmission secondary electron yield from the RSEY.

**Figure 4:**
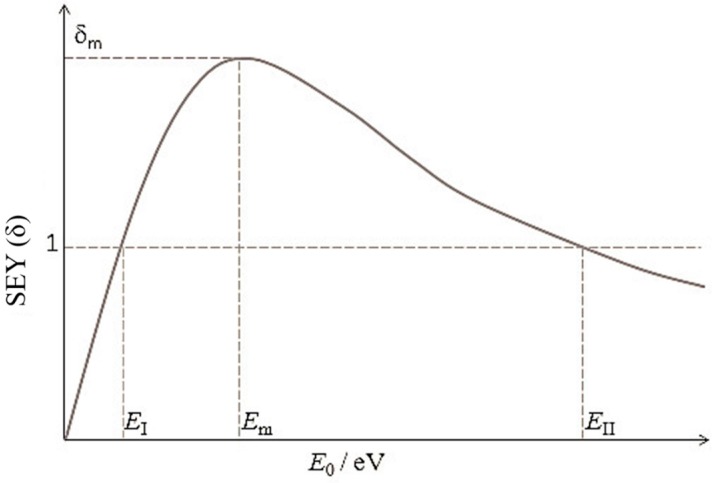
Schematic illustration for the variation of SEY with primary electron beam energy *E*_0_. The figure is reproduced from reference [15].

The most frequent way to analyze the SEE process in a reflection configuration is SEY versus the primary energy curve as presented in Figure 4 [15] with three characteristic parameters: two cross-over energies, *E*_I_ and *E*_II_, at which equals unity, and a maximum yield δ_m_ at a certain primary energy. The shape of the curve reflects the relationship between the penetration depth of the primary electrons and the escape depth of the internal secondary electrons. The penetration depth of the primary electrons, namely the depth at which the secondary electrons are generated, increases proportionally to the primary energy. As a consequence, the number of the secondaries generated also increases as a function of primary energy due to the increased number of interactions of the primary electrons with the solid.

Two important energies are the first and second crossover energies *E*_I_ and *E*_II_. When *E*_0_ < *E*_I_, the penetration depth is smaller than the escape depth and the majority of the secondary electrons can escape. However, due to the low energy of the primary electrons, a limited number of secondary electrons are generated, which results in a yield lower than one. For *E*_0_ > *E*_II_, the amount of secondary electrons is larger, but they are generated deeper inside the material. They scatter and combine before reaching the surface. As a consequence, this also leads to a yield below one. Electron multiplication occurs for *E*_I_ < *E*_0_ < *E*_II_ with a maximum yield at *E*_m_.

When *E*_0_ < *E*_m_, penetration depth is smaller than escape depth; at *E*_m_ the penetration depth becomes equivalent to the escape depth; When *E*_0_ > *E*_m_ the secondary electrons are generated so deep in the material that many lose all their energy before reaching the surface. Thus, for *E* < *E*_m_ the penetration depth of the primaries is the controlling factor whereas for *E* > *E*_m_ the escape depth of the generated electrons prevails. The net current of a solid being bombarded by energetic electrons can be zero at primary energy of *E*_I_ and *E*_II_ (δ = 1). When SEY is larger than 1, the emission of secondary electrons will leave electron vacancies inside the solids. The replenishing of vacancies by introducing some conductivities is important to avoid charge-up effects. Different approaches have been used in practice to provide electrical conductivity. In commercial dynodes, materials with high SEY, such as beryllium oxide (BeO), gallium phosphide (GaP), gallium arsenide phosphide (GaAsP), aluminum oxide (Al_2_O_3_), and magnesium oxide (MgO) are normally coated onto a conducting substrate electrode made of nickel, stainless steel, or beryllium copper (CuBe) [3,4]. Doping is also a commonly used technique for assisting recharge, for example, boron doped diamond was demonstrated to be a superb secondary electron emitter [13].

## 4. Reflection Secondary Electron Yield

The search for suitable materials used in dynodes for PMs started in the early 30s. Many insulating materials (summarized in Table 1) [16,17,18,19,20,21,22,23,24,25,26] were found to have high SEY and were successfully employed in commercial PMs based on a reflection configuration (Section 4.1). Since the 90s, diamond based materials (Section 4.2) have been another extensively studied system for SEE applications. Diamond has shown many advantages compared to traditional SEE materials. Indeed, very attractive SEE properties, including excellent transport properties and the possibility of obtaining NEA by a rather easy H termination procedure, have been demonstrated. Very recently, ALD techniques have shown great potential in synthesizing ultrathin films in the order of nanometers for novel dynodes. These materials include mostly alkali oxides, such as Al_2_O_3_ and MgO (Section 4.3). Thanks to the ability to control the film thickness and composition and surface species, ALD is expected to provide many possibilities for improved SEE materials. It should be noted that in this review only materials with favorable SEE properties for application in PM are summarized. We have set our criterion of RSEY >3.

### 4.1. Compound Insulators

A selection of materials without NEA activation process with RSEY (>3) [16,17,18,19,20,21,22,23,24,25,26,27,28] have been summarized in Table 1. The majority of the RSEY data were documented by Kenneth et al. in 1948 [25] and Joy et al. in 2008 [1]. All these materials are wide-band-gap insulators, for example, alkali halides, alkaline earth compounds with an exception of AlBe, NiCr alloys [24]. Due to the lack of detail on material characterization, the high yield of these alloys was not well understood. It is very likely caused by the oxidization of the metal elements (Al, Be or Cr) on the surface of these films.

There are some clear discrepancies (both for maximum SEY and for the primary energies where the maximum SEY was obtained) between measurements for the same type of materials. These may be the result of surface contamination, or different conditions under which the data was measured, or different assumptions when data was interpreted. For instance, for MgO studied by Whetten et al. [20], a maximum SEY as high as 24 was measured in comparison to about 3–4 measured by other researchers [18,19,20,21]. Clearly, a non-conducting material cannot sustain this level of emission for any significant period of time, since it will become positively charged and recollect its own secondary electrons. Consequently, all SEY results for insulators must be treated with caution. Therefore, the comparison of SEY of insulators prepared and measured at different conditions has to be made with great care. Even if the SEY measurements were taken at the same experimental conditions, the SEY can be strongly influenced by the composition and morphology of the materials. For example, depending on the morphology and surface treatment of the Al_2_O_3_, the SEY of one can be three times (19.0) larger than the other (about 6.4) [23]. Unfortunately, a detailed explanation of these large differences was not reported in the original paper. The uncertainties in the early literature (most of these are from the 1930s–60s) show the necessity of repeating many of the measurements systematically using modern techniques for the sake of precision and accuracy.

### 4.2. Diamond

Diamond has excellent electron emission characteristics. This is attributed to the combination of good transport characteristics, namely a large escape depth and its ability to present a NEA surface by a relatively simple surface treatment (hydrogen termination). The first evidence of the NEA of hydrogen-terminated diamond was published in 1979 by Himpsel et al. [29] as a result from their experiments with photoelectron emission from diamond (111) surfaces. The majority of the work in the last two decades has focused on investigating the influence on the SEY of the surface termination, (H, Cs, and Cs-O), boron doping level, the thickness, and morphology of the diamond and substrate materials. A wide variation of values of RSEY (from as low as below 1 to as high as higher than 100) from diamond were reported in the literature. One excellent example to demonstrate how large the SEY can be influenced by the morphology and surface conditions is the work by Yater et al. [30]. They have reported SEY values from a minimum of 3 up to as large as 132 for different crystallographic orientations with different surface treatments (single crystal (100) (111) and polycrystalline diamond with and without H or Cs terminations) [30]. Another important observation of this work is that low-energy electrons are transported and emitted very efficiently regardless of crystal orientation. However, the energy distribution and SEY varies with different crystal orientation and different surface structure and surface termination. For detailed analysis of the influences of other parameters on the SEY and a complete compilation of the values of RSEY from diamond, one should refer to the PhD thesis of R. Vaz, who has given a rather complete summary of SEE properties of diamond [14]. The focus of the present paper will mainly focus on the TSEY of diamond (Section 5).

### 4.3. ALD-Synthesized MgO and Al_2_O_3_

ALD techniques have been very recently used in fabricating SEE materials, such as Al_2_O_3_ and MgO. This section mainly summarizes the recent development of such materials from two projects: (1) the Large-Area Picosecond Photo-Detector (LAPPD) collaboration [31] for development of Micro-Channel Plates (MCPs) and (2) the MEMBrane project (which the authors are associated with) [26,32,33] for designing a novel photo detector using ultrathin transmission dynodes.

#### 4.3.1. LAPPD

The LAPPD collaboration [31] is focused on the development of large-area systems to measure the time-of-arrival of relativistic particles with, ultimately, one pico-second resolution, and for signals typical of Positron-Emission Tomography of about 30 pico-second resolution. The ongoing development of ALD enables the use of relatively inexpensive and robust borosilicate micro-channel substrates for use as MCPs [31]. The surfaces of the channels in these glass plates are functionalized to control the conductivity as well as the SEY. One of the important tasks of this collaboration is to identify and fabricate novel SEE materials with properties optimized for use in such detectors.

Jokela et al. [27,28] have performed systematic studies on the effects of film thickness and surface chemical composition on the SEY of ALD MgO and Al_2_O_3_. The samples used in the study of the effects of surface composition consisted of an Al_2_O_3_ film of thickness 11.3 nm and an MgO film of thickness 29.0 nm. The SEYs of both samples change with the increasing electron exposure time, where SEY of Al_2_O_3_ decreases while that of MgO increases. This was explained as the result of surface contamination, namely the deposition of carbon upon electron exposure. Two possible explanations were proposed: (i) the carbon compound on the material having a SEY greater than Al_2_O_3_ but lower than MgO and (ii) a different kind of bond between carbon and oxygen on the surface of the MgO (acting as a barrier for SEE) from the bond between carbon and Al_2_O_3_ surfaces.

RSEY as a function of films thicknesses was also investigated. For Al_2_O_3_ films, the maximum RSEY of 2.9 was reached when the film thickness was approximately 5 nm. For the MgO films, in contrast, the maximum SEY continues to increase with film thickness over the entire range of films, achieving a value of 6.9 for a 20 nm film. Thicker MgO samples could not be measured due to the severe charging of the films. This was proposed to be related to the differences in escape depths of secondary electrons of Al_2_O_3_ versus MgO (~230 Å versus ~410 Å [34]). The authors suggested the use of a lower electron flux may allow the measurement of thicker samples. However, the exact beam current used for this measurement is not stated with only a brief description of electron beam currents being in the range of 5–100 nA. Nevertheless, this work clearly indicated the superiority of MgO over Al_2_O_3_ for MCP applications.

Furthermore, larger SEY was observed for Ti doped MgO films, where the doping of Ti was realized by Ar ion sputtering the sample consisting of one monolayer of TiO_2_ on top of an MgO film. This was proposed to be related to the increased conductivity, the altered surface composition (affecting the work function), or the change in electronic structures. However, no clear evidence of any of these mechanisms was identified. Nevertheless, this demonstrated that the SEE properties can be further enhanced by adding other compounds or elements in low concentrations.

#### 4.3.2. MEMBrane Project

**Figure 5:**
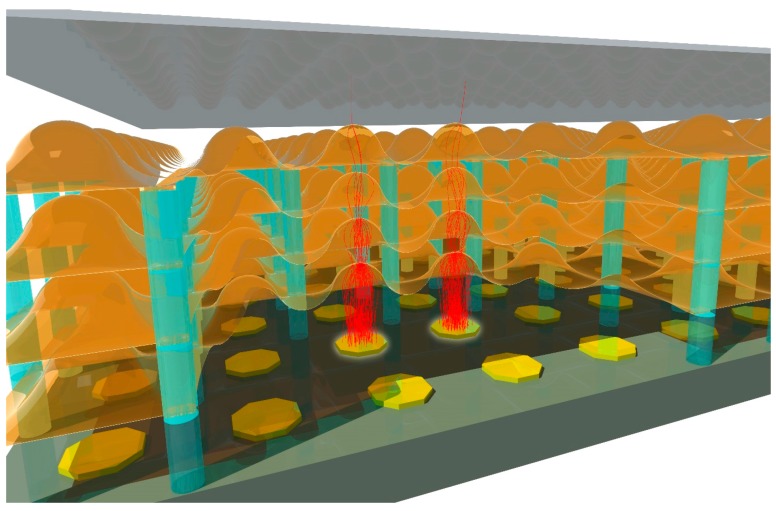
The essence of the MEMBrane project: a stack of transmission dynodes in vacuum placed on top of a CMOS pixel chip. By capping the assembly with a classical photocathode or an electron emission membrane, a photon detector “Tipsy” or charged particle tracking detector “Trixy” can be realized, respectively. The Tipsy detector, for example, is sensitive for individual soft photons (100–1000 eV), which are converted into photoelectrons in the photocathode and multiplied in the stack of transmission dynodes. The resulting electron avalanche is detected by the digital circuitry in the individual pixels of the CMOS chip.

The ultimate goal of the MEMBrane project led by H. van der Graaf [26,32,33] at Nikhef, Amsterdam is to realize a family of detectors for soft photons (100–1000 eV low energetic photons), electrons, and energetic charged particles. The core innovation of such detectors is a stacked set of curved miniature transmission dynodes in vacuum, created through Micro Electro Mechanical Systems (MEMS) fabrication techniques on top of an all-digital CMOS pixel chip (see Figure 5). The time resolution of this device can be in the order of a few pico-seconds since the electron crossing paths between two transmission dynodes is two orders of magnitude smaller than in photomultipliers, and these paths are effectively uniform straight lines towards the next transmission dynodes, with little variation. For reasons of focusing, these may have a dome shape which also deals better with possible internal mechanical stress of the membranes. Due to the thinness of the ultra-thin films, the charging up effect often found in thick insulators is limited by a conducting layer not far from (in order of 10 nm) the emission layer. Another important advantage of such a detector is that the back-flow positive ions, created in the electron multiplication process, is blocked, so degradation of photocathodes or transmission dynodes is prevented [34].

Such novel detectors have the potential to be applied in solid state, atomic, and molecular physics experiments, in medical imaging, and in commercial applications such as prompt 3D optical imaging. The main challenge in realizing such a device is the performance of the transmission dynodes, i.e., a high TSEY (>4) of the dynodes at relatively low energies (<1 keV). Various materials (Si, SiC Low-pressure chemical vapor deposition (LPCVD) silicon-rich silicon nitride, ALD Al_2_O_3_, ALD MgO) have been studied in both reflection and transmission modes using three different setups [26] for the sake of precision.

In reflection measurements, a charging effect has been observed for bulk insulating materials, such as silicon rich silicon nitride, Al_2_O_3_, and MgO. This charging effect has been reduced by increasing the Si content of the silicon rich silicon nitride, or by graphene or TiN coating on Al_2_O_3_ and MgO. The maximum RSEY of thin ALD Al_2_O_3_ (>3) and MgO (>4) (Table 1) membranes [26] were found to be comparable to the values reported within the LAPPD collaboration. Silicon rich silicon nitride (SEY > 2.5) was found to be promising because the EA was predicted to be largely reduced by light alkali metal oxide or hydrogen termination. A comparison of RSEY and TSEY of ultrathin films (in the order of ten nm) and prototype transmission dynodes will be made and discussed in Section 5.6.

## 5. Transmission Secondary Electron Yield

Exploration for materials for transmission dynodes started about 30 years later than for materials for reflection dynodes. The number of the studied materials and literature is much less than that of the reflection dynodes. The literature can be classified into three main time frames (50s–60s, 70s and 90s up to now). For each of these periods, the transmission dynodes made use of a different class of materials aiming at developing different applications.

Starting in the 50s, alkali halides, one typical example, bulk KCl (Section 5.1) and low density KCl (Section 5.2) were extensively studied for application in high speed electrons for imaging purposes [8,9,10,11]. In the 70s, research on transmission dynodes was mainly focused on application in image tubes. Bulk CsI transmission dynodes (Hamamatsu in 1972) activated by Cs [35] (Section 5.3) were found to be superior, in both TSEY and degree of degeneration when exposed to humid air, compared to low density KCl transmission dynodes. Over a similar period of time, for the same application purpose, efficient electron emission based on the concept of NEA obtained from Cs or Cs-O termination on the surface of semiconductors (GaAs [36,37] and Si [38,39]) (Section 5.4) was used in transmission dynodes. In the 90s, diamond (Section 5.5) was discovered to show NEA through hydrogen termination on the surface. This development has triggered novel applications of diamond as electron emitters in photocathodes and secondary electron emitters in PMs used in photo detectors. Although a large amount of literature has been devoted to the measurement of the RSEY of diamond, only a few reported on the TSEY of diamond [40,41,42,43]. This is mainly due to the technical challenges in fabricating ultrathin diamond films for use in transmission dynodes [12]. Therefore, the TSEY was mostly measured from film thicknesses of a few μm to hundreds of μm, where very often high primary electron energies (typically up to 10 k·eV) were applied. The latest development in exploring novel materials for transmission dynodes is the MEMBrane project (see Figure 5) [26,32,33], which started a few years ago. Within this project, ultrathin membranes with extreme thinness (in the order of 10 nm) have been made through MEMS technology (silicon rich silicon nitride) and state-of-the-art ALD technologies (Al_2_O_3_ and MgO) (Section 5.6). These ultrathin films have showed great potential for obtaining high TSEY at low primary electron energies.

### 5.1. Bulk KCl (50s and 60s)

In the 50s and 60s, bulk KCl was the preferred choice for use in transmission dynodes for high speed electron multiplication. This is mainly because KCl showed higher RSEY than other materials such as MgO, BaF_2_, and CsI (see Table 1) and, at the same time, demonstrated reproducible RSEY (controllable insulator charging effect). Photo emissive image intensifiers using KCl thin films as transmission secondary electron emitters were explored by two groups of researchers in the 1960s: Wachtel, Doughty, Anderson and Sternglass [11] and Wilcock, Emberson and Weekley from Imperial College, London [44]. The basic goal was to investigate the feasibility of developing a magnetically focused image-forming photomultiplier using dynodes consisting of thin films of a metal as an electron scatter in intimate contact with an insulator having a high secondary emission yield. The dynodes have a combination of Au (scatter)/SiO/KCl (emitter) where the Au and KCl were deposited by evaporation onto a thin supporting layer of SiO approximately 10 nm thick [12]. For dynodes without a scatter layer, a maximum TSEY of between 1 and 2 was measured with primary energy of about 5–15 keV while increasing the thickness of the KCl layer from 115 to 520 nm. The introduction of the metal scatterer (5 nm Au layer) increased the maximum TSEY to about 4.5 with similar primary energies of 5 keV. Such a scattering layer was confirmed to increase the effective thickness of the insulating layer for the incident electrons so that they deposit a greater portion of their energy in the insulator. As a result, more energy becomes available for secondary formation, while at the same time, fewer fast electrons emerge on the exit side.

It was found that the yield was increased by decreasing the thickness of the KCl and Au layers, shifting to lower energies at the same time. The data suggested that a value of about 60 nm thickness of KCl and 1.5–2 nm of Au should give a yield close to the optimum and at the same time not make the dynodes excessively fragile. The yield curve for such a dynode reached a maximum of 8.4 at primary energy of 3.2 keV. The issue of conductivity through the insulating layer was claimed to be induced by the bombardment process itself. Despite the desirable aspects, the disadvantages of such type of dynodes were the high rate of deterioration under electron bombardment and the need for relatively large overall voltages. Further development aiming at more stable materials quickly moved on to low density KCl.

### 5.2. Low Density KCl (1962)

The low density KCl dynode [12] consisted of an aluminum oxide (or silicon monoxide) supporting layer on which a thin layer of Al or Au was deposited and a secondary emitter, low density KCl. Unlike the solid film KC1 dynodes, the low density dynodes showed a significant increase in yield with the applied external collecting voltage. The results showed that, as the grid voltage is raised and thereby the internal electric field is increased, the yield reaches much larger values than can be obtained in the absence of such a field. It is seen that for a fixed incident electron energy of 300 eV, the TSEY of 2 at 4 kV increased rapidly with the increasing collecting voltage, to 9 at 9 kV and 37 at 37 kV. This was many times the yield of a bulk film. As the collecting voltage was further increased, a saturation effect became noticeable so that no further significant increase in yield takes place. From the experiments, the high yield of porous materials has a different origin compared to true secondary electron emission in conventional materials. It appears that internal electric fields in porous deposits of insulators allow one to extract a large fraction of all the secondary electrons formed by the primary ionization mechanism. In spite of the potential high gains obtainable with secondary emission image intensifiers, the employment of these dynodes was found to be limited because of the effect of the positive charging at the exit surface where the secondary electrons emitted. Indeed, it was stated: with the increase of collector voltage (>37 kV), the SEY increase and currents become unstable and a breakdown accompanied by visible localized discharges takes place [12].

### 5.3. Bulk CsI Activated with Cs (1972)

Bulk CsI transmission dynodes [35] were studied and compared with the KCl transmission dynodes [11] in Hamamatsu in Japan in 1972 for image tube applications. The transmission dynodes were in the form of a combination of Al_2_O_3_ (substrate)/Al (conductor)/CsI (emitter). The thickness of the CsI layer was found to be optimal when it was equal to or slightly larger than the range of the electrons in the layer. This gave a value of 40–60 nm for practical primary beam voltages. This is similar with those of KCl with a thickness of 60 nm [31]. As such, 20 nm of Al as a conducting layer did not reduce the TSEY. This thickness is much larger than the thickness of Au (5 nm) reported in the case of SiO/Au/KCl. A comparison of CsI transmission dynodes with the conventional KC1 transmission dynodes in terms of the TSEY and the ratio of secondary to transmitted primary electrons showed that the former was definitely superior. In addition, the TSEY could be significantly improved (almost doubled, i.e., from about 15 to about 27 at primary energy of 9 keV) by Cs activation in the emission side of the dynode. Another important advantages of using CsI as transmission dynode material is that the degree of degeneration when exposed to humid air was found to be considerably smaller than when using KCl [35].

### 5.4. Semiconductors with NEA: GaAs and Si (1970–1976)

For the same application (image tube) as the bulk CsI transmission dynodes (Section 5.3), efficient electron emission, based upon the concept of NEA, from Cs or Cs-O terminated semiconductors, such as GaAs [36,37], and Si [38,39] and other ternary Ill-V compounds, have been investigated in the same period of time. These new efficient semiconductor emitters are characterized by their long minority-carrier diffusion lengths and high electron escape probabilities. The high SEY results from the long diffusion length of the thermalized electrons at conduction band minimum before emission and the NEA of the emitting surface, where electrons can efficiently escape into the vacuum.

As a result, the RSEY of these dynodes with NEA is extremely high, 400 and 1800 (at primary energy of 20 keV) for GaAs [36] and Si [39], respectively. Following the measurement of the excellent SEY in reflection dynodes, TSEY of these thinned films (in order of a few µm) activated with Cs or O-Cs on the back side of the dynodes were measured. For, GaAs TSEY values of 30 [36] and 112 [37] (Table 2) were measured for film thicknesses of 5 and 3.5 µm with primary energies of 10 and 20 keV, respectively. The escape depth was estimated to be between 2 and 3 µm which is orders of magnitude greater than the typical hot-electron escape depth. The escape probability was between 0.14 and 0.18. The transmission secondary electron emission (TSEE) mechanism was described by a simple diffusion-transport model, where the surface-escape probability and electron-diffusion length were two important parameters.

As expected (due to the much higher RSEY of Si than GaAs), the TSEE characteristic of Si (see Table 2) was found to be even better than that of GaAs. This is due to the even larger escape depth for thermalized minority carriers of 5.5 µm compared to about 2–3 µm of GaAs. With a combination of NEA on the Si surface, the highest TSEY was 725 at 20 keV [38], primary energy for a sample with a thickness of 4–5 µm [38]. This is about 76% of the maximum RSEY (950 with primary energy of 20 keV) measured for the same sample with the same setup [38]. In addition to the EA of the surface, another important parameter, namely doping level, was claimed to play an important role in optimizing the performance [39].

### 5.5. Diamond (90s to Now)

Despite the excellent SEE characteristics of diamond, one common issue in SEY measurements in the reflection configuration is the surface degeneration, for example due to H desorption caused by bombardment of high energetic electrons. One possible method to overcome this problem is using a transmission configuration. In fact, the transmission configuration is also the appropriate approach to evaluate the escape depths of electrons in diamond. A summary of TSEY values of diamond is given in Table 2. It should be noted that all the diamond samples in Table 2 are H terminated on the backside for NEA and boron doped for good electrical conductivity.

TSEY of polycrystalline diamond samples is low (<5) using high primary-energy electrons (4–25 keV) [40,41,42,43], which was attributed to increased electron scattering at grain boundaries and longer transport distances, with low transport efficiency [43]. The diffusion lengths of single crystal diamond were found to be much larger (8.1 μm) [43] than that of polycrystalline (1.3 μm). A single crystalline diamond sample with a thickness of 8.3 µm yielded a similar TSEY (3–4) with that of a much thinner (2.5 μm) polycrystalline diamond sample [43]. One can expect a much higher TSEY for single crystalline diamond of similar thickness. However, there are several issues to consider when growing thinner single crystalline diamond films, such as poor seeding density leading to non-continuous films [14]. Using hot-filament activated chemical vapor deposition (HF-CVD), diamond as thin as 140 nm has been achieved by R. Vaz [14]. Unfortunately, the TSEY of the thin diamond film was not given in this work. Instead, transmission yields of 0.6–0.9 were measured from diamond films with thicknesses of 10–0.4 μm, under field free conditions, which after hydrogenation were increased by a factor of ~2. As demonstrated, diamond is certainly a promising candidate for transmission dynodes with high TSEY. However, efforts in fabrication of ultrathin films and optimization of their TSEY is required before any practical application in PMs.

### 5.6. Ultrathin ALD Synthesized Transmission Dynodes (after 2010s)

The TSEY of a transmission dynode consisting of a conductive titanium nitride (TiN) layer (5 nm) and a high SEE layer of ALD Al_2_O_3_ has been systematically studied by H. van der Graaf et al. within the MEMBrane project [26]. The TSEY of a thin homogeneous Al_2_O_3_ membrane reaches a maximum of 2.5 for a thickness of 10 nm, for primary electron energy of 1200 eV [26]. The TSEY, under these conditions, is 20% lower than the RSEY. This ratio of TSEY: RSEY is similar (80% versus 76%) to that found with NEA silicon transmission dynodes. For thicker membranes, 25 nm and 50 nm, lower maximum TSEY values of 2.1 and 1.8 were measured for higher primary energies of 2.3 and 3.4 keV, respectively. With a stack of transmission dynodes as mentioned above (TiN (5 nm)/Al_2_O_3_ (10 nm)), a practical vacuum electron multiplier could be made: placed on top of a Timepix-3-pixel chip, a new generic digital single electron detector is within reach (see Figure 5). There are several possibilities (Section 6), however, to increase the TSEY of the transmission dynodes, and to reduce the required energy of the incident primary electrons. This would significantly reduce both the number of needed dynodes in the stack, and the operational high voltage.

## 6. Outlook for High TSEY Materials

### 6.1. ALD

ALD is a unique thin film technology which offers the best possibility of controlling the film thickness and surface properties in a truly nanometer or sub-nanometer range. Therefore, ALD techniques provide vast opportunities for the development of transmission dynodes which relies on the precision of the thinness and surface control. A recent comprehensive survey of ALD materials by Miikkulainen et al. [45] indicated that hundreds of chemistries have been found for depositing a variety of materials during the past decades. A comparison between the most promising SEE materials with high RSEY (Table 1) and ALD materials shows many possibilities in applying ALD methods to SEE materials. This includes binary and ternary oxides (Al_2_O_3_, MgO, BaO, SrO, Li_2_O, MgAl_2_O_4_ and BaSrO_2_) and alkaline earth halides (MgF_2_, CaF_2_). In addition, wide band gap nitrides (BN, AlN, GaN) and group III-V semiconductor compounds (GaP, ZnP, GaAs, InAs) are also promising provided proper surface activation (Cs or Cs-O). Some of the processes are well established and have been in industrial use for decades, for example, Al_2_O_3_, while others are in a phase of small lab-scale production, while most of them are somewhere in between. In this section, some insights will be provided taking a few representative materials as examples.

#### 6.1.1. ALD MgO

A high TSEY is highly desired for a transmission dynode. In the case of a high TSEY, the number of the transmission dynodes needed will be accordingly small. This will greatly reduce the complexity of fabricating the transmission dynode stack. Within the LAPPD collaboration [31], the ALD synthesized MgO is reported to have a significantly higher RSEY than Al_2_O_3_ [27,28]. This is also confirmed by the recent experiments within the MEMBrane project [26]. Therefore, a systematic measurement of TSEY of ALD MgO will be the first priority of the MEMBrane project. A higher TSEY of MgO can be expected than that of Al_2_O_3_, although the optimal thickness (of the transmission dynodes) and the optimal primary electron energy may be different. The optimum thickness may be larger than that of Al_2_O_3_ (10 nm) [28] due to the larger escape depth of MgO (410 Å) than Al_2_O_3_ (~230 Å) [32]. To avoid the charging effect, a few experiments are planned. These includes (i) the use of a lower electron flux or pulsed electron beam; (ii) alternation of the conductivity of the ALD MgO by changing the composition of the ultrathin film, for instance by precisely controlling the ratio of Mg and O or doping of other components (Ti) [27]; (iii) post ALD treatment, such as a thin layer of conductive coating.

#### 6.1.2. Tunable Properties in ALD Processes

Just like other thin film growth techniques, the composition and the morphology of the films is influenced by many parameters, such as temperature, the types and ratio of the reactants, impurities, and substrate, which will therefore result in different SEE properties. Besides binary materials, ternary materials with excellent SEE properties, such as MgAl_2_O_4_, have been successfully synthesized [46]. ALD GaAs was realized in early 2016 [47]. One can expect more sophisticated materials, for example p-n junction III-V semiconductor compounds such as GaAs, will be realized by using ALD techniques in the near future.

### 6.2. NEA

Another strategy for increasing TSEY is introduction of NEA by proper surface termination. As discussed throughout this paper, applying Cs or Cs-O termination onto the emitting surface has shown to increase SEY on many materials. It should be emphasized that the limitation of using Cs activation is that the device has to be made and operate in a vacuum because Cs is extremely reactive when exposed to air. Alternative procedures, such as Li-O [48,49] and Mg-O terminations [50], have been reported to be air stable on a diamond surface. Hydrogen termination was proven to be a good choice for diamond as well [51]. One of the successful examples is the diamond amplified cathode, where a diamond transmission dynode acts as an electron amplifier. The hydrogenated diamond was demonstrated to be extremely robust with no degradation during the emission process, and with only a drop of about 50% in electron emission gain after exposure to air for six months. H and Li-O (and other light metal-oxygen) terminations were predicted to work in a similar way for Si rich silicon nitride [52,53], which showed good RSEE characterization [26].

### 6.3. Field-Assisted Emission

It is well known that the external electric field at the surface of a metal or a semiconductor can significantly modify the potential barrier and induce field emission of electrons [54]. There is a region in which this electric field causes an increase of the yield before “cold” field emission occurs: this is described as sub-threshold emission [55]. Dramatic increase of TSEY (from 1 to 200) of SOI/SiO_2_/Si_3_N_4_ triple layer transmission dynode by increasing the external field from 100 V to 300 V was reported by Qin et al. [56]. The multiplication was realized by subthreshold Fowler-Nordheim field emission. The device architecture demonstrated [56] allows for an easy realization of stacked electron and other particle detectors and for probing electronic, photonic, or phononic excitations of thin semiconductor membranes. The positive effect of an external field on the TSEY was also demonstrated in one of the first transmission dynodes, made of low-density porous KCl materials [12]. It was reported to have a TSEY of 37 with an external extracting field of about 37 kV (see Table 2). The SEE mechanism of such materials is claimed to be different in nature compared to bulk crystalline material [57]. As a result of charging inside the porous material, strong electric fields appear inside the cavities of the material. Secondary electrons that are generated inside the material will cause a new cascade of electrons [57]. There are reports on the use of MgO covered carbon nanotubes as a SEE material. The SEE mechanism is comparable to that of porous materials. The sharp tips of the covered carbon nanotubes will cause strong electric fields near the tips: gain of 103 has been reported [58]. Preliminary results from the MEMBrane project also indicate that the positive effect of an accelerating field has an intrinsic positive extracting effect on the transmitted secondary electrons of ultrathin ALD Al_2_O_3_ films [26].

### 6.4. Design of the Conductive Layer for Transmission Dynodes

A conductive layer can be applied to the impact side of the transmission dynodes [26,35], as well as to both sides, in a sandwiched construction with the emission material in-between. This material should provide sufficient conductivity for the optimum thickness of the TSEY materials and, in the meantime, preserve as much primary electron energy as possible by avoiding large amounts of electron–electron scattering. Metals are not well suited to be used as the conductive layer for transmission dynodes since, for instance, a 1 keV electron loses practically all of its energy passing through a layer of 2 nm thick Au. TiN and graphene have been explored within the MEMBrane project, where TiN is preferred [26]. This is probably because TiN has similar properties as the so-called transparent conducting oxides (TCOs). They all have rather large band gaps (less electron-electron scattering) and are transparent but also conductive (good electron/hole mobility). TCOs are usually compound semiconductors, where the nonmetal part is oxygen. As n-type TCOs are of special importance for thin-film solar cell production, indium-tin oxide and the reasonably priced aluminum-doped zinc oxide have been widely studied [59]. These materials may be suitable for use as a conductive layer for ultrathin transmission dynodes. A dedicated study of conducting materials should be performed to select materials with optimal properties, namely good electron/hole conductivity and minimal electron–electron scattering. This can be done by computational modeling, either using Monte Carlo simulations [60] or self-consistent drift–diffusion-reaction simulations [61].

## 7. Conclusions

High RSEYs (>3) have been observed in many materials like alkali halides, alkaline earth compounds (mostly oxides), diamonds, and semiconductors. Such materials either have a large band gap (preventing electron–electron scattering) or excellent electron transport properties (semiconductors). Many of these materials showed significant SEY enhancement by surface treatments which reduces the EA of the secondary electron emitting surface. The surface treatments can be done by H (Cs) termination for diamond and Cs (Cs-O) termination for semiconductors (GaAs, Si etc.).

One essential requirement for a transmission dynode is a high TSEY. Another important feature of a transmission dynode is the thickness of the material. The ideal thickness for obtaining a high TSEY, with a low primary electron energy (100–1000 eV), is in the order of 10 nm. However, most of the materials (diamond, Si, and GaAs) can only be made with a thickness of a few micrometers. Therefore, most of the TSEY measurements summarized in this review have been obtained using extremely high-energy primary electrons (a few keV to larger than 10 keV).

The ALD techniques, allowing atomic precision, show great promise and open up many possibilities in synthesizing novel materials and in fabricating novel structures for use in transmission dynodes. Most recent results indicate the suitability of ALD synthesized Al_2_O_3_. However, better performance can be expected for MgO since it was demonstrated to have higher RSEY than Al_2_O_3_. There are several possibilities to further optimize the performance of such transmission dynodes. These include improving the conducting layer, exploring new ALD recipes, applying NEA surfaces, and applying external fields.

## Figures and Tables

**Table 1:** Materials with RSEY (Figure 2) greater than 3. δ_m_ is the maximum SEY found in the SEY as a function of primary energy curve (Figure 4). *E*_m_ is the primary energy (in eV) where the maximum SEY was found.

Materials	δ_m_	*E*_m_ (eV)	References
LiF	5.6		[16]
NaF	5.7		[16]
NaCl	6.0	600	[17]
NaCl	6.8		[16]
KCl	7.5		[16]
KCl	13.4	1800	[18]
RbCl	5.8		[16]
CsCl	6.5		[16]
NaBr	22.5	1500	[18]
NaBr	24.0	1800	[19]
NaBr	6.2		[16]
KBr	13.4	1800	[18]
KI	10.8	1500	[18]
KI	10.0	1600	[20]
KI	5.5		[16]
CaF_2_	3.2		[16]
BaF_2_	4.5		[16]
MgO	4.0	400	[21]
MgO	24.3	1300	[20]
MgO	3.3	750	[22]
MgO	3.6	1250	[22]
BaO	4.8	400	[21]
Al_2_O_3_ (polished sapphire)	7.8	650	[23]
Al_2_O_3_ (sapphire)	6.4	750	[23]
Al_2_O_3_ (lucalox)	19.0	1300	[23]
Al_2_O_3_ (polished lucalox)	6.4	250	[23]
Al_2_O_3_ Mg_2_SiO_4_	4.9	650	[23]
Al_2_O_3_ Mg_2_SiO_4_ (<1% Ba)	4.2	800	[23]
MgAl_2_O_4_	4.7	600	[23]
MgAl_2_O_4_ (0.2%–1% Ca)	4.1	500	[23]
AlBe	4.5	600	[24]
NiCr	3.7	500	[24]
BeO	3.4	2000	[25]
BaO SrO	8.0	1500	[25]
BaO SrO	5.0–12.0	1400	[25]
Al_2_O_3_	4.8	1300	[25]
ALD Al_2_O_3_ (12.5 nm)	3.6	400	[26]
ALD MgO (50 nm)	4.4	600	[26]
ALD Al_2_O_3_ (20 nm)	3.7	380	[27]
ALD Al_2_O_3_ (11 nm)	3.3	350	[28]
ALD MgO (20 nm)	5.2	800	[27]
ALD MgO (29 nm)	9.6	550	[28]

**Table 2:** Transmission dynodes or materials with TSEY (δ) higher than 1 (see Figure 2 and Section 3 for illustration and definition of TSEY, respectively.) and E_0_ is the energy of the primary electron.

Transmission Dynodes	Thickness	δ	E_0_ (k·eV)	References
SiO	10 nm	1.4	2	11
SiO/Au/KCl	115 nm (Au 5 nm)	4.2	5	11
	60 nm (Au 2 nm)	8.4	3.2	
SiO/KCl	115 nm	1.7	2.5	11
	260 nm	1.8	4.5	
	520 nm	1.7	7.0	
Porous Al/KCl ^a^	19 µm	2	0.3 (4)	12
		9	0.3 (9)	
		24	0.3 (24)	
		37	0.3 (37)	
Al_2_O_3_/Al/CsI	60 nm/20 nm/70 nm	15	9	35
Al_2_O_3_/Al/CsI (Cs)	60 nm/20 nm/70 nm	27	9	35
GaAs	5 µm	30	10	36
GaAs	3.5 µm	112	20	37
Si	4–5 µm	725	25	3838
	10 µm	~600	25	
Si	3 µm	~550	22	39
	5 µm	~560	21	39
	10 µm	~600	25	39
PCD ^b^ diamond	2 µm	4	5	40
	5 µm	2.5	20	
f-NCD ^c^ diamond	0.15 µm	1	4	41
		5	7	
	2.5 µm	1	15	
		3	18	
PCD diamond	2.5 µm	4	25	42
SCD ^d^ (100) diamond	8.3 µm	3–4	20	43
PCD diamond	2.5 µm	3–4	20	43
HF-CVD ^e^	0.4 µm	1.2	1.3	14
TiN/Al_2_O_3_	5 nm/10 nm	2.5	1.25	26
	5 nm/25 nm	2.1	2.3	
	5 nm/50 nm	1.8	3.4	

^a^ the value in the braces is the value of external field (KV) applied during the measurement was taken; ^b^ PCD = polycrystalline diamond; ^c^ f-NCD = facetted monocrystalline diamond; ^d^ SCD = single crystal diamond; ^e^ HF-CVD = hot-filament activated chemical vapor deposition.
